# Skull destruction from intracranial metastasis arising from pulmonary squamous cell carcinoma: a case report

**DOI:** 10.1186/1752-1947-7-28

**Published:** 2013-01-24

**Authors:** Imran Kader, Michael Strong, Mathew George

**Affiliations:** 1 University of Newcastle Bachelor of Medicine Program, University of Newcastle, University Drive, Callaghan, NSW 2308, Australia; 2 North West Cancer Centre, Hunter New England Area Health Service, Johnston St, Tamworth, NSW 2340, Australia

**Keywords:** Destruction of bone, Metastasis, Squamous cell carcinoma of the lung

## Abstract

**Introduction:**

Squamous cell carcinoma of the lung represents 30% of all non-small cell lung carcinomas. It arises from dysplasia of squamous epithelium of the bronchi and is strongly associated with cigarette smoking. Squamous cell carcinoma of the lung is known to produce metastases in the brain parenchyma.

**Case presentation:**

We present the case of an 80-year-old indigenous Australian man with an unusual presentation of metastatic carcinoma of the lung. The case demonstrated a squamous cell carcinoma of the lung with an intracranial metastatic lesion destroying the parietal bone and extending into the extracranial soft tissue. A visible deformity as a result of the metastasis was evident on physical examination and computed tomography demonstrated extensive bone destruction.

**Conclusion:**

The authors were unable to find a case of this occurring from a squamous cell carcinoma of the lung anywhere in the world literature. The case report demonstrates an unusual disease presentation with a rare intracranial metastasis invading through the skull.

## Introduction

**Figure 1:**
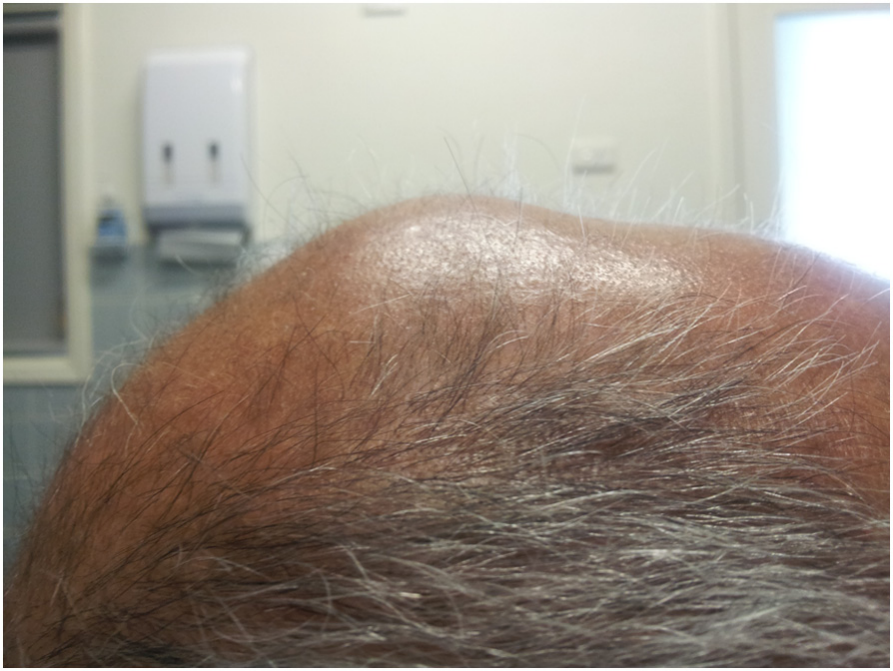
**Metastatic brain lesion on examination. **This image is a lateral photograph of the patient’s skull demonstrating the palpable swelling found on physical examination. It demonstrates the extracranial extension of the intracranial metastatic lesion.

Lung cancer is the most common cause of death from cancer and contributes significantly to the burden of disease [1,2]. Squamous cell carcinoma of the lung (SqCC) represents 30% of all non-small cell lung carcinomas (NSCLC) [1]. SqCC arises from dysplasia of the squamous epithelium of the bronchi and is conventionally defined via the histopathologic features of keratinization and intracellular bridges [3]. SqCC is strongly associated with cigarette smoking. Over 50% of patients with NSCLC have disseminated disease at the time of diagnosis [2]. The brain is a frequent site of metastases for carcinoma of the lung and lung cancer is responsible for approximately 50% of all brain metastases. Over half of all brain tumors are the result of metastatic disease [4]. Of brain metastases, 80% originate from the hemispheres of the cerebrum and most are well demarcated with a capsule. A minority of lesions may demonstrate infiltrative growth [5]. Metastatic brain lesions are responsible for significant morbidity and mortality and have a dismal prognosis (Figure 1) [4,6]. The clinical features of brain metastases vary depending on the location of the lesion and may be due to either paraneoplastic or direct effects [7]. The most common complaint of brain metastases is headache, found in 24% to 53% of patients. Other common symptoms include altered mental status, focal weakness, seizures and ataxia [8].

## Case presentation

**Figure 2:**
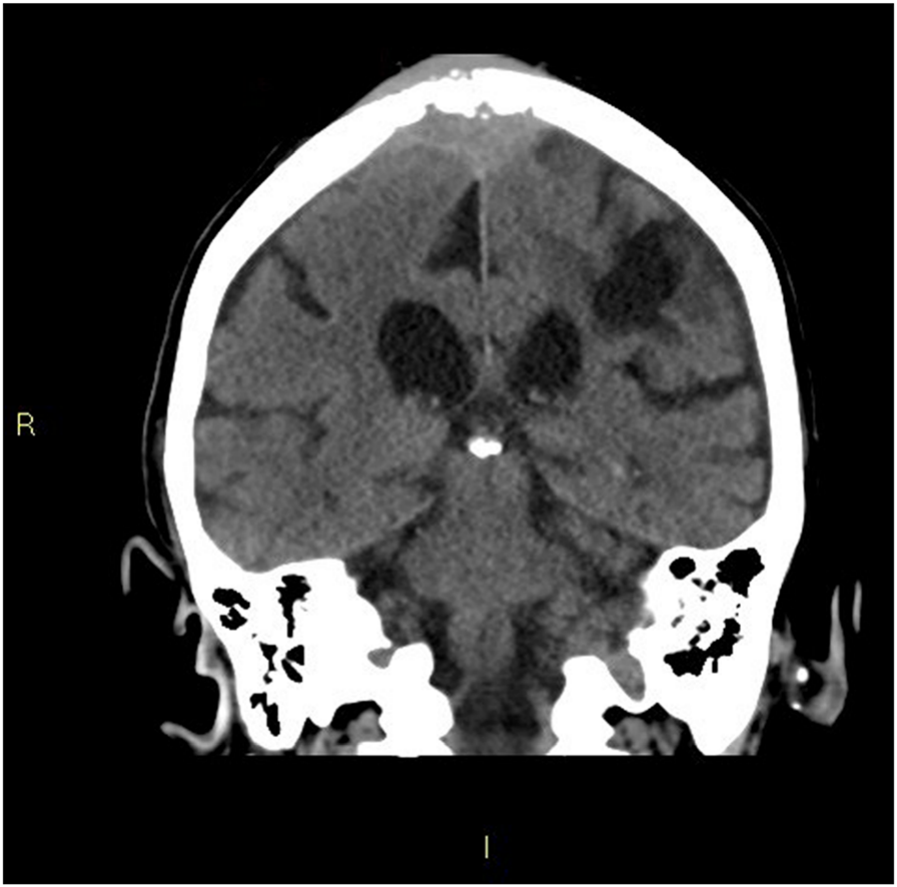
**Chest X-ray. **This is a posteroanterior chest X-ray taken of the patient at the time of diagnosis. It demonstrates the opacity in the left upper lobe of the lung consistent with lung cancer.

**Figure 3:**
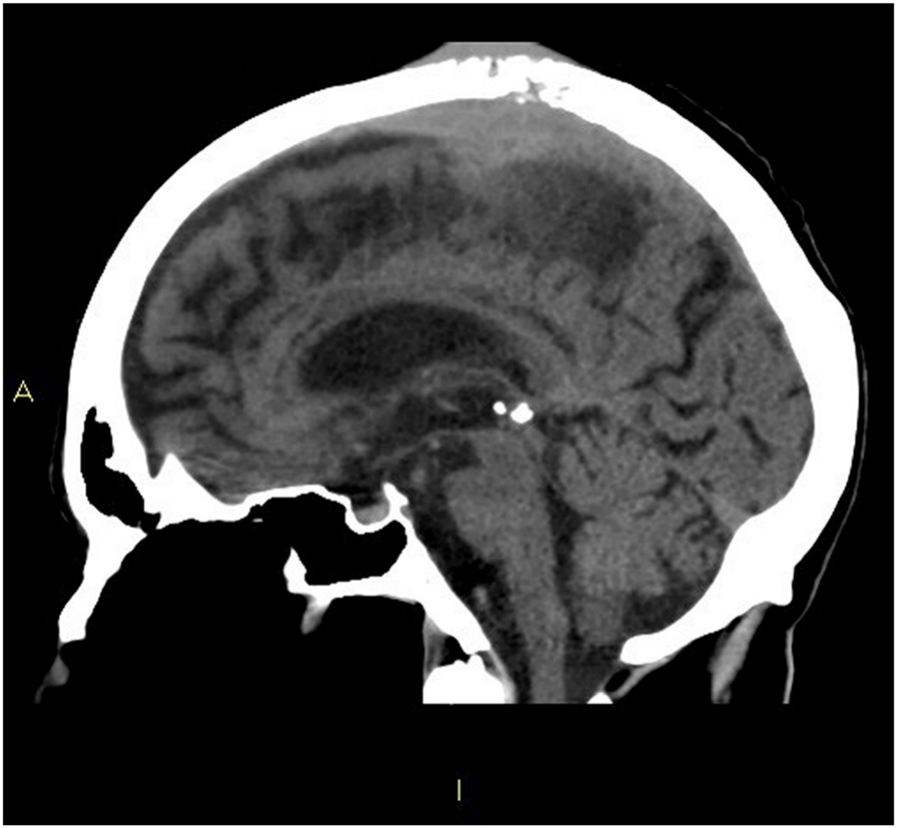
**Coronal head computed tomography slice. **This image is a coronal slice of the head computed tomography performed on the patient. It demonstrates a single intracranial metastasis with destruction of the parietal bone and extension of the lesion into the extracranial soft tissues.

**Figure 4:**
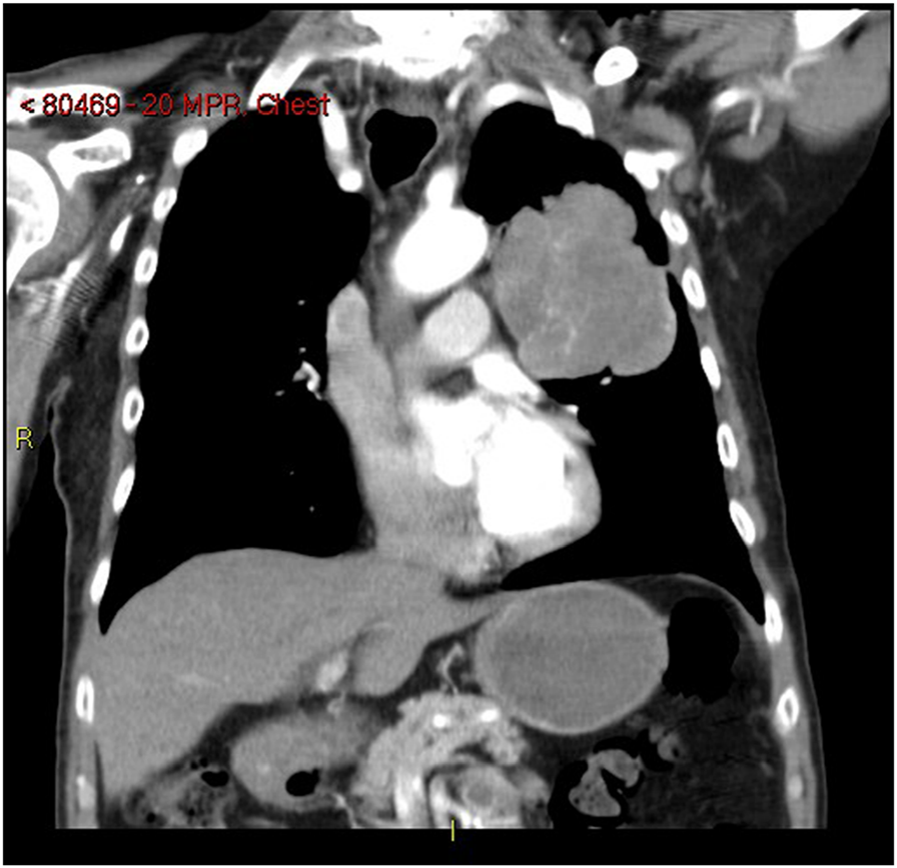
**Coronal chest computed tomography slice. **This image is a coronal slice of the chest computed tomography taken of the patient. It demonstrates a large squamous cell carcinoma of the lung in the left upper lobe.

**Figure 5:**
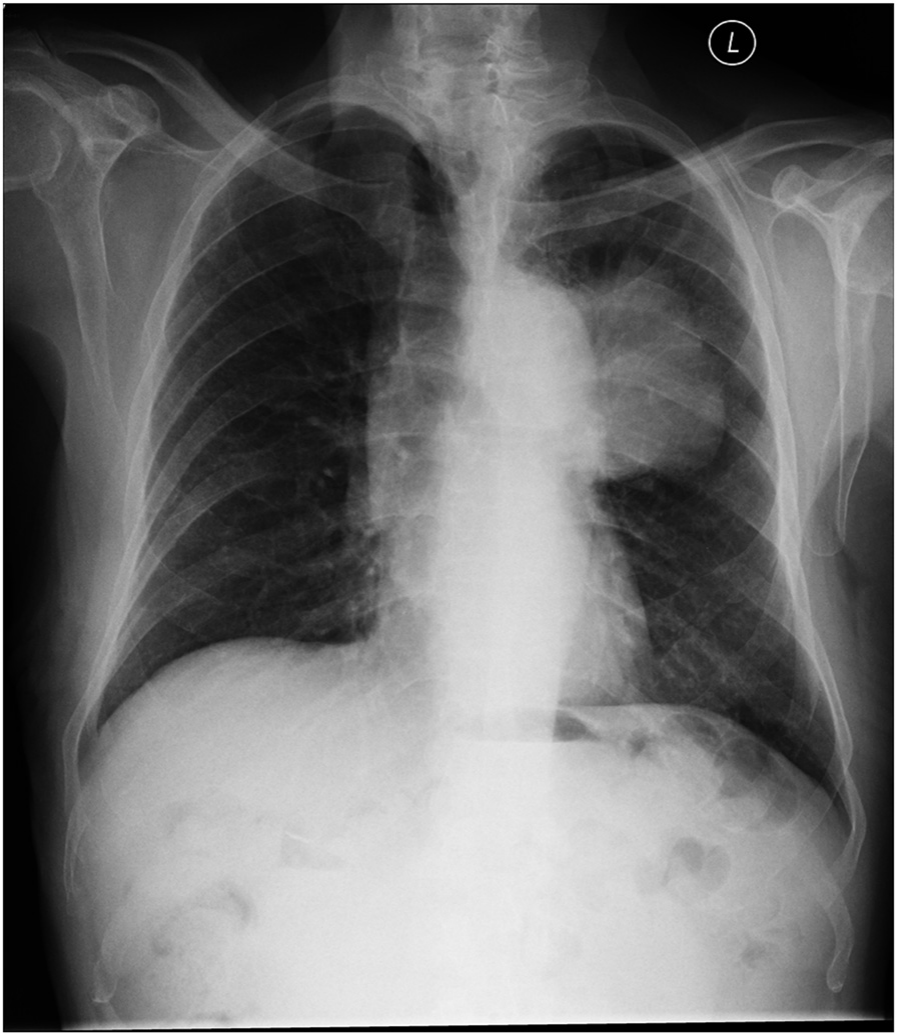
**Sagittal head computed tomography slice. **This image is a sagittal slice of the head computed tomography performed on the patient. It demonstrates the intracranial metastasis.

An 80-year-old indigenous Australian man presented to his general practitioner complaining of a headache, persistent cough with minimal hemoptysis, weight loss and night sweats over a period of two months. He was an ex-smoker of cigarettes with an 80-pack per year history of tobacco use. His past medical history was unremarkable. A physical examination revealed a palpable swelling of the scalp overlying the parietal bone. He had no history of trauma to the head. His general practitioner requested a chest X-ray that demonstrated a large mass in the left parahilar region extending into the anterior segment of the left upper lobe (Figure 2). It was reported as highly suspicious for a primary neoplasm and thought to be a bronchogenic carcinoma. The man was referred to our institution for a medical oncology opinion. A contrast-enhanced computed tomography (CT) of his chest and a non-contrast CT of his head were requested (Figure 3). The chest CT demonstrated the presence of a mass in the anterior left upper lobe measuring 8.78×7.40×7.79cm extending to and invading the anterior and lateral pleura (Figure 4). The mass extended to the left hilum and invaded the anterior mediastinum and contacted the aortic arch. There was evidence of left upper lobe bronchial obstruction and compression of second order bronchi. No lymphadenopathy was noted. The head CT demonstrated a destructive lesion along the vertex of the parietal bone measuring 14mm transverse and 44mm anteroposterior. There were soft tissue components extending intracranially and extracranially. The intracranial component had a maximum thickness of nine mm and had a mass effect on the superior sagittal sinus (Figure 5). An area of possible long-standing gliosis with calcification was noted in the left temporoparietal lobe. The CT findings were consistent with a metastatic lesion originating from the meninges intracranially, with some involvement of the brain parenchyma and extending through the parietal bone to the extracranial soft tissues. A bronchoscopy was performed with bronchial washings, bronchial brushings and a biopsy for histopathology was taken. The biopsy contained scanty mucous-like tissue which did not withstand processing. The bronchial washings and bronchial brushings contained a moderate number of abnormal cells arranged singly and in clusters with pleomorphic, hyperchromatic nuclei, coarse chromatin and a small volume of dense cytoplasm consistent with SqCC. Epidermal growth factor receptor (EGFR) testing was not performed. The patient declined palliative radiotherapy and was managed medically with 8mg of dexamethasone daily and opioid analgesia.

## Discussion

SqCC is typically a centrally located lung carcinoma with four variants recognized by the World Health Organization 2004 classification: clear cell, small cell, papillary and basaloid [1,9]. It is staged most commonly according to the seventh edition of the TNM classification of malignant tumors [10]. The stage of the tumor is an important determinant of prognosis and the key determinant to guiding treatment [11]. This case represents a rare occurrence of an intracranial metastatic SqCC lesion producing destruction of the parietal bone to invade beyond the skull and into the extracranial soft tissue. It also highlights an unusual presentation of lung cancer. It is extremely rare for intracranial metastases from lung carcinomas to produce destruction of bone. The authors were unable to find a published case of this occurring from an SqCC anywhere in the literature. Destruction of bone from a metastatic brain lesion originating from a primary lung carcinoma has been reported by Foco *et al*. in the only published case of adenocarcinoma metastaticum that produced destruction of the frontal bone [12]. Secondary metastases from Ewing’s sarcoma, carcinoma of the prostate, breast, thyroid and neuroblastoma have been reported to lead to destruction of the bone on rare occasions [13,14]. The mechanism of invasion of bone tissue from metastatic lesions has not been fully elucidated. Bone tissue is highly resistant to destruction and most of the information regarding bone destruction is derived from studies focusing on breast cancer, multiple myeloma and prostate cancer, which metastasize to the bone rather than directly invade it [15]. Injuries to the periosteum have been implicated in making the bone more prone to malignant tumor invasion [16]. Studies that looked at oral SqCCs directly invading the mandible proposed a role for interleukin (IL)-6 in the activation of osteoclastic activity and subsequent bone resorption [17]. A recent model by Roato *et al*. concluded that NSCLC bone-invading cells produce IL-7, which is known to promote osteolytic lesions [18]. All of these pathological processes may be involved in this case. The prognosis of patients with stage four SqCC is extremely poor with a median survival of four months [10]. Patients with brain metastases from any primary source have a median survival of one month from diagnosis if not treated [4]. As a result, treatment in this population should be focused primarily on palliation. Management of patients with intracranial metastatic lesions extending extracranially is poorly studied. There is a demonstrated role for surgical resection followed by whole brain radiation therapy with doses of 30Gy to 40Gy in patients with a single intracranial metastatic lesion, particularly if the lesion appears capsulated and there are no extracranial lesions. The proposed benefits include improved neurocognitive function, elimination of mass effects and removal of the source of perifocal edema. Open surgical or stereotactic radiosurgery can be employed [5]. Palliative radiotherapy without surgery has also been used with or without dexamethasone. A case report by Velnar *et al*. outlined surgical removal of a meningioma, portions of the skull and portions of soft tissue in a tumor that invaded through the skull, however, this was for a benign lesion [19]. Platinum-based chemotherapy agents, such as cisplatin and carboplatin, are reported to have a 29% response rate against intracranial metastatic brain lesions arising from SqCC and may be employed in patients with well-controlled neurologic symptoms. A 10% to 38% response to gefitinib and erlotinib was demonstrated in brain metastases from NSCLC that express EGFR [6]. This patient was offered palliative radiotherapy but declined due to the travel and time commitments associated with accessing the treatment. Surgical resection was not considered an option. He opted for palliative medical management only.

## Conclusion

The authors find this to be an interesting and unique case due to the rare phenomenon of an intracranial metastasis from a SqCC invading through the skull and into the extracranial soft tissues, which has not previously been described in the literature. It also highlights an unusual presentation of SqCC and raises questions over the most appropriate course of management of patients with intracranial metastases that invade through the skull.

## Consent

Written informed consent was obtained from the patient for publication of this case report and accompanying images. A copy of the written consent is available for review by the Editor-in-Chief of this journal.

## Abbreviations

CT: Computed tomography; EGFR: Epidermal growth factor receptor; IL: Interleukin; NSCLC: Non-small cell lung carcinoma; SqCC: Squamous cell carcinoma of the lung.

## Competing interests

The authors declare that they have no competing interests.

## Authors’ contributions

IK wrote the Introduction, Discussion and Conclusion sections. MS wrote the Case report section and performed the literature review. MG is the patient’s treating physician, supplied the images and reviewed the article. All authors read and approved the final manuscript.

## Authors’ information

IK is a final year medical student at the University of Newcastle, Australia. MS is a final year medical student at the University of Newcastle, Australia. MG is a consultant medical oncologist at Tamworth Base Hospital, Australia.
